# Comparison of cross-sectional HIV incidence assay results from dried blood spots and plasma

**DOI:** 10.1371/journal.pone.0172283

**Published:** 2017-02-23

**Authors:** Katherine E. Schlusser, Christopher Pilcher, Esper G. Kallas, Breno R. Santos, Steven G. Deeks, Shelley Facente, Sheila M. Keating, Michael P. Busch, Gary Murphy, Alex Welte, Thomas Quinn, Susan H. Eshleman, Oliver Laeyendecker

**Affiliations:** 1 Department of Medicine, Johns Hopkins University School of Medicine, Baltimore, MD, United States of America; 2 Department of Medicine, School of Medicine, University of California at San Francisco, San Francisco, CA, United States of America; 3 University of São Paulo, São Paulo, Brazil; 4 Grupo Hospitalar Conceicao, Porto Alegre, Brazil; 5 Blood Systems Research Institute, San Francisco, California, United States of America; 6 Public Health England, London, United Kingdom; 7 The South African DST/NRF Centre of Excellence in Epidemiological Modelling and Analysis (SACEMA), Stellenbosch University, Stellenbosch, South Africa; 8 Laboratory of Immunoregulation, Division of Intramural Research, National Institute of Allergy and Infectious Diseases, NIH, Baltimore, MD, United States of America; 9 Department of Pathology, Johns Hopkins University School of Medicine, Baltimore, MD, United States of America; Centers for Disease Control and Prevention, UNITED STATES

## Abstract

**Background:**

Assays have been developed for cross-sectional HIV incidence estimation using plasma samples. Large scale surveillance programs are planned using dried blood spot (DBS) specimens for incidence assessment. However, limited information exists on the performance of HIV cross-sectional incidence assays using DBS.

**Methods:**

The assays evaluated were: Maxim HIV-1 Limiting Antigen Avidity EIA (LAg-Avidity), Sedia HIV-1 BED-Capture EIA (BED-CEIA), and CDC modified BioRad HIV-1/2 Plus O Avidity-based Assay (CDC-BioRad Avidity) using pre-determined cutoff values. 100 matched HIV-1 positive plasma and DBS samples, with known duration of infection, from the Consortium for the Evaluation and Performance of HIV Incidence Assays repository were tested. All assays were run in duplicate. To examine the degree of variability within and between results for each sample type, both categorical and continuous results were analyzed. Associations were assessed with Bland Altman, R^2^ values and Cohen’s kappa coefficient (ĸ).

**Results:**

Intra-assay variability using the same sample type was similar for all assays (R^2^ 0.96 to 1.00). The R^2^ values comparing DBS and plasma results for LAg-Avidity, BED-CEIA, and CDC-BioRad Avidity were 0.96, 0.94, and 0.84, respectively. The concordance and ĸ values between DBS and plasma for all three assays were >87% and >0.64, respectively. The Bland-Altman analysis showed significant differences between plasma and DBS samples. For all three assays, a higher number of samples were classified as recent infections using DBS samples.

**Conclusions:**

DBS and plasma sample results were highly correlated. However, when compared to plasma, each assay performed somewhat differently in DBS at the lower and higher ends of the dynamic range. DBS samples were more likely to be classified as recently infected by all three assays, which may lead to overestimation of incidence in surveys using performance criteria derived for plasma samples.

## Background

HIV incidence is the number of new infections that occur over a period of time in a particular population [1]. Measurements of HIV incidence are used to study the HIV/AIDS epidemic, determine populations and geographic areas at higher risk for infection, and evaluate the efficacy of interventions targeted towards these higher risk groups [2, 3]. Serological assays are one of the methods utilized to screen populations for HIV incidence. Almost all data currently available on the performance of incidence has been generated on stored serum or plasma samples [4–7]. Very little information is currently available on the use of dried blood spots (DBS) for cross sectional incidence testing. However, it would be beneficial if DBS samples could be used with these assays, particularly when large studies are conducted in resource-poor settings. The drawbacks to using plasma and serum include the invasive nature of drawing blood, the processing required to separate plasma and serum from whole blood, and the need for cold transport and storage. In contrast, dried blood spots are collected through a minimally invasive procedure and can be stored and transported at ambient temperature up to 14 days after collection [8].

Very little data on the performance of HIV incidence assays on DBS samples has been published in peer reviewed publications, independent of developers of these incidence assays. One previous study, presented at a scientific conference [9], directly examined the results of Maxim HIV-1 Limiting Antigen Avidity EIA (LAg-Avidity) from matched DBS and plasma. They determined that there was a high correlation between sample type for both continuous and categorical results. One other published study explored the use of DBS on an HIV incidence assay using the Calypte HIV-1 BED Incidence EIA. However, the DBS results were not compared to matched plasma results so it is unclear if the DBS results obtained are comparable to the results that would have been generated using plasma [10]. To further investigate the use of DBS samples to screen populations for HIV incidence, we tested matched plasma and DBS samples on LAg-Avidity, Sedia BED HIV-1 Incidence EIA (BED-CEIA), and CDC-BioRad Avidity [4, 11, 12].

## Methods

### Ethics statement

This study was approved by the Institutional Review Board of the University of California at San Francisco School of Medicine (IRB# 10–02365, Title: The HIV Panels Project and development and evaluation of assays to detect recent HIV infection and estimate HIV incidence) and the Johns Hopkins School of Medicine eIRB2 (IRB# NA00004380, Title: HIV Prevention Trials Network: Laboratory Center). All trial and cohort studies were conducted according to the ethical standards set forth by the institutional review boards of the participating institutions and the Helsinki Declaration of the World Medical Association. All participants provided written informed consent. This report includes analysis of stored samples and data from those studies.

### Sample characteristics and storage

100 matched plasma and DBS samples were obtained from the Consortium for the Evaluation and Performance of HIV Incidence Assays (CEPHIA). DBS samples were prepared at three different testing sites, one in the United States, and two in Brazil. The site in the United States contributed 75 samples while the sites in Brazil contributed 25 samples total. DBS samples were prepared by pipetting 50 μl of whole blood per spot from a fresh tube of venous whole blood in EDTA onto Whatman® 903 Protein Saver Cards. The volume of whole blood used to make blood spots is important, since it has been shown that the volume of serum obtained from a 6 mm punch increases with increasing spot volume if the hematocrit is kept constant [13].

All samples were positive for HIV and had a known duration of infection. For the purposes of this study, those samples from individuals known to be infected < 1 year were classified as ‘recent’ while those samples from individuals known to be infected >1 were classified as ‘long term’. Other sample characteristics are shown in Table 1. The plasma samples were stored at −80°C and the DBS samples were stored at −20°C.

**Table 1:** Characteristics of matched plasma and DBS samples.

Characteristic	N
*Duration of Infection*	
>1 year	75
<1 year	25
*Viral Load*	
>10,000	31
400–10,000	21
<400	40
Unknown	8
*CD4 Cell Count*	
>500	58
200–500	33
50–199	2
<50	0
Unknown	7
*On ART*	
No	76
Yes	24
*Country*	
Brazil	25
United States	75

### Sample preparation

When preparing the DBS for elution, 6 mm punches were taken from each sample, which contained approximately 13 μl of whole blood. Forceps were used to transfer the sample punches into the appropriate titer tubes. Titer tubes are 1.2 mL polypropylene tubes that do not have caps and are disposable. They come in a rack of 96 tubes. After each transfer, the forceps were wiped with a 70% ethanol solution and allowed to dry before being used again. Furthermore, six blank punches were made between each DBS sample punch (6 mm diameter) in order to reduce the possibility of contamination. These blank punches were not eluted.

DBS samples were eluted overnight at 4°C without agitation for all three assays. The following elution volumes were used: LAg-avidity: 500 μl; BED-CEIA: 400 μl; CDC-BioRad Avidity: 300 μl. The sample diluent used for each assay was provided by the respective manufacturer. A previous study found that at a 55% hematocrit, a 6 mm punch from a 50 μl spot contains approximately 5.5 μl of serum[13]. Using this estimation of the volume of serum in a 6 mm punch from a 50 μl spot at a 55% hematocrit, it can be determined that less than 2 μl of sample is entered into each test. This volume is calculated by taking into account the volume of sample diluent used for elution and the amount of eluate required for each assay. The required eluate volume for LAg-Avidity, BED-CEIA, and CDC-BioRad Avidity is 100 μl. Thus, the estimated volume of serum added per well to each assay is as follows: LAg-Avidity: 1.10 μl; BED-CEIA: 1.38 μl; and CDC-BioRad Avidity: 1.83 μl. These estimated values demonstrate that less serum is added to the above assays when using DBS samples compared to traditional serum samples.

For CDC-BioRad Avidity, the incident DBS controls were made from an HIV seroconversion panel purchased from Zeptometrix Corporation (Catalog No. HIV 9081, panel members 9081–03 and 9081–04) and the prevalent controls were made from a plasma sample collected from an individual known to have a long-term HIV infection. To prepare the DBS controls for CDC-BioRad Avidity, control plasma samples were mixed with red blood cells at a 40% hematocrit; 50 μl of this mixture was then spotted onto Whatman® 903 Protein Saver Cards. The cards were dried overnight and then placed in sealed bags with desiccant packs and humidity indicators and stored at -20°C prior to use. The DBS controls used with LAg-Avidity and BED-CEIA were provided in their respective kits[14].

### Sample testing

All sample testing was performed in a single, centralized laboratory by one technician. Samples were tested in duplicate on LAg-Avidity, BED-CEIA, and CDC-BioRad Avidity. The manufacturer’s protocol was followed for both the plasma and the DBS testing done using LAg-Avidity and BED-CEIA. The CDC-BioRad Avidity testing was completed using the protocol optimized by the CDC for both sample types. The BioRad Avidity protocol optimized by the CDC provides a result known as an avidity index (AI). Avidity index values are ratios of the optical density (OD) values obtained from two different test wells for each sample. During the antibody dissociation step of the assay, one well is treated with BioRad wash buffer and one well is treated with 0.1 M diethylamine (DEA). The DEA reagent dissociates antibodies that are weakly bound to the target antigens. The OD value from the DEA-treated well is divided by the OD value from the wash buffer-treated well and multiplied by 100 to obtain the AI, which is expressed as a percentage[4]. Further description of this assay modified for use in DBS can be found in the recently published manuscript by Wei and colleagues[15]. Duplicate samples were run on the same plate for BED-CEIA and LAg-Avidity and on different plates for CDC-BioRad Avidity. Matched plasma and DBS samples were run on the same plate for Sedia BED and CDC-BioRad Avidity. Conversely, the matched plasma and DBS samples were run on different plates for Maxim LAg-Avidity because Maxim manufactures separate kits for plasma and DBS testing that have different lot numbers. Each time a DBS sample was run on an assay a single 6 mm punch was used.

### Statistical methods

Both continuous and categorical results were analyzed to determine the degree of variability within and between sample type results. Correlation between continuous results was evaluated using Pearson’s correlation coefficient (r), R^2^, and Bland-Altman plots, while correlation between categorical results was assessed using Cohen’s kappa coefficient (ĸ). The cutoff for determining recent and long-term infection was 1.5 OD-n for LAg-Avidity, 0.8 OD-n for BED-CEIA, and 30% AI for CDC-BioRad Avidity. These cutoffs were established using serum or plasma samples. Statistical analyses were performed in STATA version 11 (StataCorp, College Station, TX).

## Results

### Correlation between continuous results

**Fig 1:**
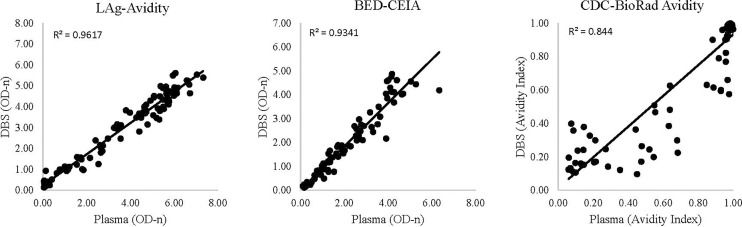
Correlation of results from matched plasma and DBS samples tested with LAg-Avidity, BED-CEIA, and CDC-BioRad Avidity.

Variability within sample type was low for both plasma and DBS. The R^2^ value was 0.99 for plasma samples run on LAg-Avidity and BED-CEIA and 0.96 for plasma samples run on CDC-BioRad Avidity. Similarly, R^2^ was 1.00, 0.99, and 0.97 for DBS samples run on LAg-Avidity, BED-CEIA, and CDC-BioRad Avidity, respectively (Table 2). These R^2^ values are based on the replicates of each sample. For variability between plasma and DBS results the R^2^ values were 0.96, 0.93, and 0.84, for LAg-Avidity, BED-CEIA, and CDC-BioRad Avidity, respectively (Fig 1).

**Table 2:** R^2^ values for replicate plasma and DBS samples tested with LAg-Avidity, BED-CEIA, and CDC-BioRad Avidity.

	LAg-Avidity	BED-CEIA	CDC-BioRad Avidity
Plasma	0.99	0.99	0.96
DBS	1.00	0.99	0.97

When using a cutoffs of 1.5 or 3.0 OD-n with the LAg-Avidity assay, the average difference of OD-n values indicates that both plasma and DBS samples had greater variability above the assay cutoffs than below the assay cutoffs. For plasma samples, using a cutoff of 1.5 OD-n, replicates had an average difference of 0.18 (SD: 0.17) for values above the cutoff and an average difference of 0.03 (SD: 0.04) for values below the cutoff. Using a cutoff of 3.0 OD-n, plasma replicates had an average difference of 0.19 (SD: 0.18) above the cutoff and 0.08 (SD: 0.10) below the cutoff. For DBS samples, using a cutoff of 1.5 OD-n, replicates had an average difference of 0.11 (SD: 0.09) for values above the cutoff and an average difference of 0.06 (SD: 0.05) for values below the cutoff. Using a cutoff of 3.0 OD-n, DBS replicates had an average difference of 0.11 (SD: 0.09) for values above the cutoff and 0.07 (SD: 0.05) for values below the cutoff.

Comparing the three assays, the average differences of the OD-n values of plasma replicates run on LAg-Avidity and BED-CEIA were 0.14 (SD: 0.16) and 0.08 (SD: 0.07), respectively. In contrast, the average difference of the OD-n values of matched plasma and DBS samples was 0.74 (SD: 0.55) for LAg-Avidity and 0.26 (SD: 0.33) for BED-CEIA. The average difference of the AI values of plasma replicates run on CDC-BioRad Avidity was 0.05 (SD: 0.07) while the average difference of the AI values of matched plasma and DBS samples was 0.09 (SD: 0.12) (Table 3).

**Table 3:** Average differences (standard deviations) of replicate plasma samples and matched plasma and DBS samples tested with LAg-Avidity, BED-CEIA, and CDC-BioRad Avidity.

	LAg-Avidity	BED-CEIA	CDC-BioRad Avidity
Plasma (replicates on same plate)	0.14 (0.16)	0.08 (0.11)	n/a
Plasma (replicates run on different plates)	n/a	n/a	0.05 (0.07)
DBS versus plasma	0.74 (0.55)	0.26 (0.33)	0.09 (0.12)

**Fig 2:**
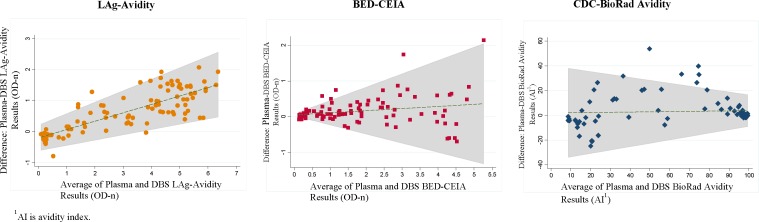
Bland-Altman plots of results obtained from testing matched plasma and DBS samples with LAg-Avidity, BED-CEIA, and CDC-BioRad Avidity.

The Bland-Altman plots showed significant differences between plasma and DBS results (Fig 2). For the LAg-Avidity assay there was an increase in the plasma values relative to the DBS values as the average OD-n increased. When the OD-n was 2 the DBS value was 0.5 OD-n less than the plasma value. At the high OD-n values there was a full unit difference between DBS and plasma. For the BED-CEIA, DBS-plasma differences were minimal at the low OD-n (values < 1.0) and greater at the high values, but differences occurred in both directions. For the CDC-BioRad Avidity assay the variation between DBS and plasma was greatest at the lower values: among values with an AI of 40% or less, DBS and plasma measurements differed by up to 20% of the AI value.

### Correlation between categorical results

**Fig 3:**
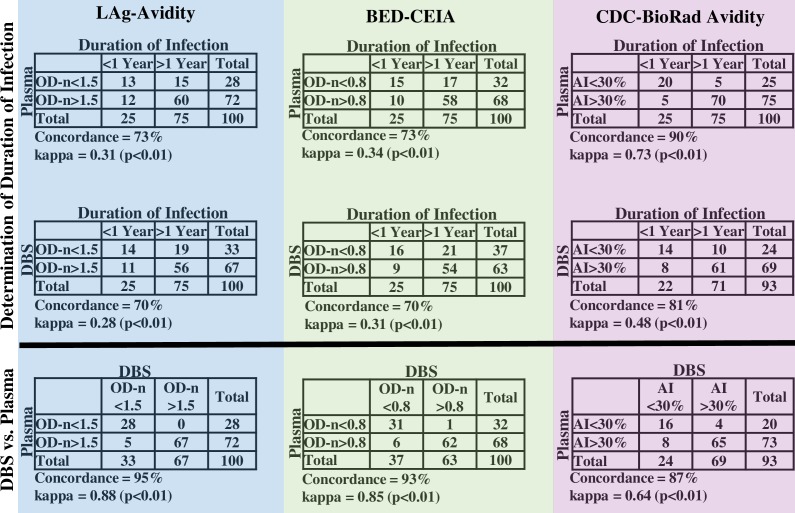
Comparison of categorical results obtained from epidemiologic data and results obtained by testing plasma and DBS samples. Results from LAg-Avidity (highlighted in blue), BED-CEIA (highlighted in green), and CDC-BioRad Avidity (highlighted in pink).

Compared to plasma, a higher proportion of DBS samples were classified as recent for all three assays (Fig 3). The concordance values between DBS and plasma for LAg-Avidity, BED-CEIA, and CDC-BioRad Avidity were 95%, 93%, and 87%, respectively. The ĸ values between DBS and plasma for LAg-Avidity, BED-CEIA, and CDC-BioRad Avidity were 0.88, 0.85, and 0.64, respectively. When comparing categorical assay results to clinically determined categories, plasma samples had higher concordance and ĸ values than DBS for all assays, and the ordinal rank of assays for concordance with plasma was the same for plasma and for DBS. The CDC-BioRad Avidity had the fewest misclassified samples for both plasma (concordance: 90%; ĸ: 0.3) and DBS (concordance: 81%; ĸ: 0.48). Seven DBS samples were excluded from the CDC-BioRad Avidity analysis due to protocol guidelines, which state that any sample that has a wash well OD value below the assay cutoff should not have an AI calculated.

## Discussion

DBS and plasma results were highly correlated for the assays that were examined. However, DBS samples were more likely than plasma to be classified as recent for all three assays, suggesting that using DBS may result in an over-estimation of incidence in surveys using performance criteria derived for plasma samples. Thus, it may be necessary to adjust the DBS protocols for these assays or make appropriate changes to the cutoffs when DBS samples are used. Moreover, the Bland-Altman analysis demonstrates that the variation between the continuous results obtained from plasma and DBS differs by assay. This analysis also demonstrates that matched plasma and DBS samples have more variability at the higher end of the range of OD-n values for LAg-Avidity, which reflects the trend among both plasma and DBS replicate average differences. For both sample types, the average difference in LAg-Avidity OD-n values is greater above 1.5 OD-n compared to below 1.5 OD-n.

There were several limitations to this study. Firstly, the sample size was relatively small. Also, due to the finite sample amount it was not possible to compare inter-technician or inter-laboratory variability between the plasma and DBS results. Also, it was unfeasible to examine the reproducibility between lot numbers of a particular kit. In the future, it would be beneficial to screen larger numbers of matched plasma and DBS samples for HIV incidence using the same assays to confirm the results that are presented above. A possible reason for the variation in performance is that we did not control for hematocrit and punch location bias [16]. Punch location can be easily controlled given sufficient sample size available on the DBS card so that partial-spot punches can be avoided. The additional testing needed to control hematocrit concentrations would greatly increase the cost and complexity of performing DBS based incidence assays. However, previous research demonstrated that when blood spot volume remains constant, the volume of serum obtained from a 6 mm punch varies with the hematocrit of the original blood sample [13]. Moreover, antiretroviral treatment and viral suppression are associated with false-recent incidence assay test results. This reflects down-regulation of anti-HIV antibodies when the level of circulating antigen is reduced [17, 18].

In our study we used a simple classification based on one year infection as the definition of recent infection. Further investigation is warranted to determine if the mean duration of recent infection (MDRI) and the false recent rate (FRR) are influenced by the source (plasma vs. DBS) of the sample, as these are the true performance metrics of a cross-sectional incidence assay or algorithm[19]. These studies require larger sample sizes to accurately determine if there are significant differences in MDRI and FRR.

## Supporting information

S1 Data All data used in analyses. OD is optical density, OD-n is normalized optical density, and AI is the avidity index. “Wash” refers to the sample well that had wash buffer added to it during the dissociation step of CDC-BioRad Avidity and “DEA” refers to the sample well that had DEA added to it during this assay step. The country abbreviations are as follows: United States (US) and Brazil (BR). For the column titled “ARV Treated at Draw,” FALSE means that the individual was not on ARV treatment when the sample was drawn and TRUE means that the individual was on ARV treatment. For the column titled “Duration of Infection,” 0 means that the individual was infected for less than 1 year and 1 means that the individual was infected for more than 1 year.
(XLSX)
